# Hepatitis B virus infection among inpatients of a psychiatric hospital of Mexico

**DOI:** 10.1186/1745-0179-1-10

**Published:** 2005-07-29

**Authors:** Cosme Alvarado Esquivel, Miguel Ángel Arreola Valenzuela, Miguel Francisco Mercado Suárez, Francisco Espinoza Andrade

**Affiliations:** 1 Facultad de Medicina, Universidad Juárez del Estado de Durango. Durango, Dgo. México; 2 Hospital Psiquiátrico Dr. Miguel Vallebueno. Secretaria de Salud. Durango, Dgo. México; 3 Hospital Regional Dr. Ignacio Téllez. IMSS. Durango, Dgo. México; 4 Hospital de Nuevo Ideal. Secretaria de Salud. Nuevo Ideal, Dgo. México

## Abstract

**Background:**

The epidemiology of the hepatitis B virus (HBV) infection in psychiatric patients from developing countries is poorly studied. Therefore, we sought to determine the frequency of HBV surface antigen (HBsAg) and HBV surface antibody (HBsAb) serological markers of HBV infection in a population of patients of a psychiatric hospital in Durango City, Durango, Mexico, and to determine whether there are any epidemiological characteristics of the subjects associated with the infection.

**Methods:**

Out of 150 patients of the psychiatric hospital of Durango City, 99 were examined for HBsAg and HBsAb by AUSZYME MONOCLONAL (Abbott Laboratories, Abbott Park, IL, USA) assay and AUSAB (Abbott Laboratories, Abbott Park, IL, USA) assay, respectively. Epidemiological data from each participant was also obtained. For comparison purposes, 2505 blood donors were examined for HBsAg seropositivity.

**Results:**

Out of the 99 patients studied, twelve showed serological evidence of HBV infection (12.1%); 7 of them (7.1%) were positive for HBsAg, and 5 (5.1%) were positive for HBsAb. Out of the 2505 blood donors, 2 (0.0008%) were HBsAg positive. Seropositivity to HBV markers was associated with an age of 45 years and older (OR = 4.27; 95%CI = 1.02–18.78). Other characteristics as gender, number of hospitalizations, duration of the last hospitalization, and clinical diagnosis were not associated with seropositivity to HBV infection markers. Patients showed a significantly higher HBsAg seropositivity than blood donors (p < 0.0000001)

**Conclusion:**

HBV was found to be an important infectious agent in the Mexican psychiatric inpatient population studied. Health care strategies for prevention and control of HBV infection in psychiatric hospitals should pay special attention to patients aged forty-five years and older.

## Background

Hepatitis B virus (HBV) infections are an important cause of morbidity and mortality worldwide. More than 400,000,000 persons are chronically infected by HBV [1,2]. Moreover, the virus is responsible for more than 300,000 cases of liver cancer every year and for similar numbers of gastrointestinal haemorrhage and ascites [2]. In the United States, chronic HBV infection is responsible for about 5,000 annual deaths from cirrhosis and hepatocellular carcinoma [1]. In Latin America, statistical estimates of HBV-related morbidity showed that greater than 150,000 acute HBV cases occur per year [3].

Reports indicate that patients with mental illnesses have a high prevalence of HBV infection [4-7], and the prevalence of this infection in psychiatric patients varies substantially in different countries. For instance, in a Brazilian study, 22.4% of the psychiatric patients studied were positive for HBV infection [8]. In European countries, a study carried out in Spain showed that HBV seropositivity in mentally handicapped adults was 81.3% [9]. In contrast, in Northern Ireland, only 4.5% of the residents of an institution for the mentally handicapped carried any HBV marker [10]. In the U.S.A., a study performed on people with severe mental illness showed a 23.4% prevalence rate of HBV infection [11]. In Asian countries, a Taiwanese study reported that 18.1% of institutionalized psychiatric patients were positive for HBV surface antigen (HBsAg) [12]. Furthermore, a study performed on psychiatric inpatients in Singapore showed that 12.7% were positive for HBsAg, 63.4% were positive for HBV surface antibody (HBsAb), and 69% were positive for HBV core antibody [13].

There is a lack of information concerning the epidemiology of HBV infection in psychiatric patients in Mexico. Therefore, we conducted a cross-sectional, prospective, descriptive survey in order to determine the frequency of HBsAg and HBsAb in a population of inpatients of a psychiatric hospital of Durango, a northern Mexican city. In addition, we sought to determine whether any epidemiological data of the patients correlated with HBV infection.

## Methods

### Study population

This study was performed in a psychiatric hospital of Durango City, Durango, Mexico during the years 1995 and 1996. A sample size calculation was performed based on the reported prevalence of HBV infection in a comparable Latin-American population [8]. A 99% confidence level sample size of 74 patients was obtained. From a total of 150 inpatients, 105 were randomly chosen and invited to participate in the study. Out of the 105 chosen inpatients, 3 were excluded since they were severely ill and unable to decide to participate in the study. Of the remaining 103 chosen inpatients, 99 agreed to participate. This study was evaluated and accepted by the Institutional Ethical Committee. A written informed consent was obtained from all the individuals participating in the study. For comparison purposes of HBsAg seropositivity, we studied 2505 blood donors that attended a local blood bank during the same study period.

### Serology for HBsAg and HBsAb

Sera, from the 99 patients were analyzed for HBsAg and HBsAb by AUSZYME MONOCLONAL (Abbott Laboratories, Abbott Park, IL, USA) assay and AUSAB (Abbott Laboratories, Abbott Park, IL, USA) assay, respectively. Sera, from blood donors, were analyzed for HBsAg only by using AUSZYME MONOCLONAL (Abbott Laboratories, Abbott Park, IL, USA) assay.

### Epidemiological data

Epidemiological data including age, gender, diagnosis, number of hospitalizations, duration of last hospitalization, and history of drug use, blood transfusion, sexual promiscuity, and surgery from all ninety-nine patients studied were obtained. Data was obtained from the patients, medical examination records, and informants. Classification of mental illnesses was performed according to the ICD-10 criteria [14].

### Statistical analysis

Sample size calculation as well as analysis of results were performed with the aid of the software Epi Info 6. To assess the association between the characteristics of the subjects and the disease, the crude odds ratio with a 95% exact confidence interval was used. We calculated the exact confidence interval because the cell value (number of cases) was less than 5 in some comparisons. For comparison of the frequencies among the groups, the Fisher exact test was used. A *p *value of less than 0.05 was considered significant.

## Results

### Serology for HBsAg and HBsAb

Out of the ninety-nine patients studied, twelve (12.1%) showed serological evidence of HBV infection. HBsAg was detected in seven patients (7.1%) while HBsAb was detected in five patients (5.1%).

Out of the 2505 blood donors studied, 2 (0.0008%) were positive for HBsAg. This frequency of HBsAg seropositivity in patients was significantly higher than that found in blood donors (p < 0.0000001).

### Epidemiological data

Of the ninety-nine psychiatric patients studied, thirty were women and sixty-nine were men. The mean age was 39.4 years (range: 18 to 80 years). We were able to trace the number of hospitalizations and duration of the last hospitalization in ninety-three out of the ninety-nine patients. Of these patients, eighty two had been hospitalized once while eleven patients had been hospitalized from two to five times. The mean duration of the last hospitalization was 7.5 years (range: 0.1 to 36 years). Patients suffered from a number of mental illnesses including schizophrenia (F20–29, n = 35), organic delusional [schizophrenia-like] disorder (F06.2, n = 23), mental retardation (F70–79, n = 18), alcohol abuse (F10, n = 5), drug dependence (F19, n = 4), dementia of the Alzheimer type (F00, n = 2), vascular dementia (F01.0-1, n = 2), depression (F32, n = 2), personality disorder (F60.9, n = 1), and hyperkinetic disorders (F90, n = 1). In six patients diagnosis was not available. Of the participants, four (4.0%) had risk factors for hepatitis B: three had received blood transfusions, and one had sexual promiscuity; eleven more patients were drug users, but they denied the use of intravenous drugs. Similarly, patients denied any history of surgery. The twelve patients with HBV positive markers had a mean age of 42.7 years (range: 18 to 65 years), nine were male and three female. Separately, HBsAg positive patients had a mean age of 39.6 years (range: 18 to 65 years), and HBsAb positive patients had a mean age of 47.0 years (range: 25 to 56 years). Eleven patients had been hospitalized once, and one patient three times. The mean duration of their hospitalizations was 9.7 years (range: 0.1 to 35 years). Out of the twelve patients, two were drug users. Furthermore, of the 12 patients with HBV positive markers, three suffered from organic delusional [schizophrenia-like] disorder, four schizophrenia, three mental retardation, one vascular dementia, and in one patient the diagnosis was not available. Table 1 shows the main epidemiological data of the patients and its relation to HBV seropositivity. The patients with negative results for HBV markers had a mean age of 38.9 years (range: 18 to 80 years), of whom, sixty were male and twenty-seven were female. We were able to obtain the number of hospitalizations in eighty-one out of the eighty seven HBV negative patients. Of these, seventy-one of them had been hospitalized once, while ten had been hospitalized from two to five times. The mean duration of their hospitalization was 7.1 years (range: 0.1 to 36 years). Out of the eighty-seven HBV negative subjects, nine were drug users, three had received blood transfusions and one had sexual promiscuity. Of these patients, thirty-one suffered from schizophrenia, twenty organic delusional [schizophrenia-like] disorder, fifteen mental retardation, five alcohol abuse, four drug dependence, two dementia of the Alzheimer type, one vascular dementia, two depression, one personality disorder, one hyperkinetic disorder, and in five patients, the diagnosis was not available. Table 2 shows a comparison between the clinical diagnosis in patients with positive HBV markers and patients with negative HBV markers.

**Table 1:** Comparison of epidemiological characteristics of the psychiatric patients with positive HBV markers and the psychiatric patients with negative HBV markers.

Epidemiological Characteristics	Patients with positive markers N = 12 (%)	Patients with negative markers n = 87 (%)^c^	OR^a^	95% ECI^b^
Age				
less than 45 years	5 (41.7%)	58 (71.6%)		
45 years and older	7 (58.3%)	23 (28.4%)	4.27	1.02–18.78
				
Gender				
Female	3 (25.0%)	27 (31.0%)		
Male	9 (75.0%)	60 (69.0%)	1.35	0.30–8.33
				
Number of hospitalizations				
One	11 (91.7%)	71 (87.7%)		
More than one	1 (8.3%)	10 (12.3%)	0.65	0.01–5.45
				
Duration of last hospitalization				
Equal or less than 5 years	5 (41.7%)	46 (56.8%)		
More than 5 years	7 (58.3%)	35 (43.2%)	1.84	0.46–7.96
				
History of blood transfusion	0 (0.0%)	3 (3.4%)	0	0.00–18.39
				
History of drug use	2 (16.7%)	9 (10.3%)	1.73	0.16–10.25

^a^OR: Odds ratio.

^b^ECI: Exact confidence interval.

^c^Some data were available in only 81 subjects.

**Table 2:** Comparison of predominant clinical diagnosis of the psychiatric patients with positive HBV serological markers and the psychiatric patients with negative HBV serological markers.

Clinical Diagnosis	Patients with positive markers	Patients with negative markers	OR^a^	95% ECI^b^
Organic delusional disorder	3 (27.3%)	20 (24.4%)	1.16	0.18–5.46
				
Schizophrenia	4 (36.4%)	29 (35.4%)	1.04	0.21–4.52
				
Mental retardation	2 (18.2%)	13 (15.9%)	1.18	0.11–6.71

^a^OR: Odds ratio.

^b^ECI: Exact confidence interval.

## Discussion

In this study, 12.1% of the psychiatric inpatients showed seropositivity to either HBsAg or HBsAb. In addition, we found a remarkable and significantly higher HBsAg seropositivity in patients than in blood donors. The frequency of HBsAg seropositivity found in our psychiatric population was also much higher than those reported in Mexican blood donors [15-17]. When comparing our results with those reported in similar psychiatric populations of other countries, our frequencies were only comparable with that found in northern Ireland [10]. However, our figure was much lower than those reported in Brazil [8], the U.S.A. [11], Spain [9], Singapore [13], and Taiwan [12]. A likely explanation for this finding could be differences in the characteristics of the patients studied. For instance, intravenous drug use, an important risk factor, which was not present in our patients studied. Similarly, other risk factors such as blood transfusion and sexual promiscuity were only present in three and one of our patients, respectively. When the characteristics of our patients were analyzed for any association with HBV infection, we observed that an age forty-five years and older were associated with HBV infection (OR = 4.27; 95% CI = 1.02–18.78). A possible explanation of this finding is that the longer the life of a subject, the higher the possibility to contract the virus. Age of psychiatric patients has been correlated with HBV infection [6], but our result conflicts with the lower HBsAg carrier rate in the aged found by Chang et al [12]. Other characteristics of the patients such as gender, clinical diagnosis, number of hospitalizations, duration of last hospitalization, history of drug use, blood transfusion, sexual promiscuity, and surgery were not associated with HBV infection. HBV infection was slightly lower in females than males in our study, and this result agrees with that reported by Chang et al [12]. With respect to clinical diagnosis, risk groups for HBV infection include chronic schizophrenia patients [18], and institutionalized psychotic patients [19]. Nevertheless, frequencies of schizophrenia patients and psychotic patients were not statistically different in HBV positive and HBV negative patients of our study. Likewise, although a long duration of hospitalization has been correlated with HBV infection [9], our HBV positive patients had a slightly longer but not statistically significant mean duration of hospitalization than the HBV negative patients (9.7 years vs. 7.2 years). This result agrees with that reported by Gmelin et al [12].

A high prevalence of HBV infection does not only occur in patients but also in personnel of psychiatric hospitals [20-22]. Therefore, our results highlight the need to take action to prevent and control HBV infection in psychiatric hospitals in Mexico. Immunization is by far the single most effective prevention measure for HBV infection [23]. Hepatitis B vaccine led to a decline in the incidence of hepatitis D virus [24], and reduces incidence of liver cancer [2]. Our results may be used as a guide when the hepatitis B vaccination is being considered for use in a psychiatric hospital. Besides vaccination programs, further recommendations include health education, HBV serologic screening of patients and personnel, and the elimination of overcrowding in psychiatric hospitals.

## Conclusion

HBV is an important infectious agent in psychiatric inpatients in Durango, Mexico. Nevertheless, the incidence of HBV infection found is lower than the majority of those reported in other countries. HBV infection was associated with an age of forty-five years and older.

## Competing interests

The author(s) declare that they have no competing interests.

## Authors' contributions

Author 1 (CAE) conceived and designed the study protocol, participated in the coordination and management of the study, performed the data analysis and wrote the manuscript. Author 2 (MAAV) obtained the general data of the patients, and performed the clinical evaluation of the patients.

Author 3 (MFMS) analyzed blood donors, and performed the data analysis.

Author 4 (FEA) obtained the general data of the patients, and performed the data analysis.
